# Effectiveness of a Hybrid Exercise Program on the Physical Abilities of Frail Elderly and Explainable Artificial-Intelligence-Based Clinical Assistance

**DOI:** 10.3390/ijerph19126988

**Published:** 2022-06-07

**Authors:** Deyu Meng, Hongzhi Guo, Siyu Liang, Zhibo Tian, Ran Wang, Guang Yang, Ziheng Wang

**Affiliations:** 1 Chinese Center of Exercise Epidemiology, Northeast Normal University, Changchun 130024, China; mengdy539@nenu.edu.cn (D.M.); liangsy427@nenu.edu.cn (S.L.); wangr489@nenu.edu.cn (R.W.); 2 Graduate School of Human Sciences, Waseda University, Tokorozawa 169-8050, Japan; hz-guo@moegi.waseda.jp; 3 Sports Marketing Research Group, Sports College, Dankook University, Gyeonggi-do, Yongin-si 16890, Korea; 72201545@dankook.ac.kr; 4 Advanced Research Center for Human Sciences, Waseda University, Tokorozawa 169-8050, Japan

**Keywords:** frail, Tai Chi, strength training, endurance training, machine learning

## Abstract

Background: Due to the low physical fitness of the frail elderly, current exercise program strategies have a limited impact. Eight-form Tai Chi has a low intensity, but high effectiveness in the elderly. Inspired by it, we designed an exercise program that incorporates eight-form Tai Chi, strength, and endurance exercises, to improve physical fitness and reverse frailty in the elderly. Additionally, for the ease of use in clinical practice, machine learning simulations were used to predict the frailty status after the intervention. Methods: For 24 weeks, 150 frail elderly people completed the experiment, which comprised the eight-form Tai Chi group (TC), the strength and endurance training group (SE), and a comprehensive intervention combining both TC and SE (TCSE). The comparison of the demographic variables used one-way ANOVA for continuous data and the chi-squared test for categorical data. Two-way repeated measures analysis of variance (ANOVA) was performed to determine significant main effects and interaction effects. Eleven machine learning models were used to predict the frailty status of the elderly following the intervention. Results: Two-way repeated measures ANOVA results before the intervention, group effects of ten-meter maximum walking speed (10 m MWS), grip strength (GS), timed up and go test (TUGT), and the six-minute walk test (6 min WT) were not significant. There was a significant interaction effect of group × time in ten-meter maximum walking speed, grip strength, and the six-minute walk test. Post hoc tests showed that after 24 weeks of intervention, subjects in the TCSE group showed the greatest significant improvements in ten-meter maximum walking speed (*p* < 0.05) and the six-minute walk test (*p* < 0.05) compared to the TC group and SE group. The improvement in grip strength in the TCSE group (4.29 kg) was slightly less than that in the SE group (5.16 kg). There was neither a significant main effect nor a significant interaction effect for TUGT in subjects. The stacking model outperformed other algorithms. Accuracy and the F1-score were 67.8% and 71.3%, respectively. Conclusion: A hybrid exercise program consisting of eight-form Tai Chi and strength and endurance exercises can more effectively improve physical fitness and reduce frailty among the elderly. It is possible to predict whether an elderly person will reverse frailty following an exercise program based on the stacking model.

## 1. Introduction

With a rapidly aging global population, the number of elderly with frailty is expected to significantly increase [1,2]. Frailty is defined as a clinical state, which means when exposed to endogenous or exogenous stressors, there will be an increased probability of negative health-related event occurrence (including disability, hospitalizations, institutionalizations, and death) [3]. Fried et al. [4] proposed five clinical criteria: unintentional body weight loss, exhaustion, inactivity, slowness, and weakness. The elderly are considered frail when three or more phenotypes are met. There is a significant impact on the lives of individuals when compared to the non-frail elderly: maximum oxygen uptake in the frail elderly is 25.5% lower than in the non-frail elderly [5]; meanwhile, sarcopenia is closely related to the physical component of frailty syndrome [6]. Frailty exerts enormous strain, not only on individuals, but also on health systems and the social economy. The frail elderly account for approximately 11.0% of the population in high-income countries [7], with up to 53.0% of the frail elderly requiring long-term care [8]. The average annual healthcare costs of the frail elderly in the United States, Netherlands, and Australia range between USD 7000 and 17,500, which is much higher than the cost of the non-frail elderly [9,10,11]. Nonetheless, frailty is not routinely captured in hospital coding systems, which means that contrary to special cases such as stroke, the frail elderly remain anonymous at the system level [12]. Additionally, such a large elderly population may have been neglected or minimized. To sum up the above, it is critical to address the health concerns of the frail elderly.

Fortunately, frailty is bidirectional and may be prevented, postponed, or even reversed with specific interventions and personalized health strategies [13]. Physical exercise is an effective method to increase skeletal muscle mass, neuromuscular control, and immunity, all of which contribute to the prevention and even reversal of frailty [14,15,16]. Furthermore, strength and endurance training have been shown to have a positive impact on specific neuromuscular and cardiorespiratory adaptations [17]: endurance training increased maximum oxygen uptake by 12.0% and lower limb strength in the frail elderly [18]. Strength training reduces fall risk by 22.0% in the frail elderly [19]. Tai Chi is a form of traditional physical and mental training that ranges in intensity from low to medium intensity, depending on the training style, posture, and duration. Studies have shown that Tai Chi training improves health-related biomarkers, and for a better body balance, Tai Chi gait recruits more lower limb muscles than conventional gait [20,21]. Therefore, Tai Chi may be an economic and effective exercise program for improving balance [22], strength [23], and aerobic endurance [24,25], as demonstrated by the fact that after one year of Tai Chi training, the maximum oxygen uptake of the elderly increased by 18.7%, and even the risk of falls in the frail elderly reduced by 47.5% [26].

While the aforementioned works achieved good performance for the frail elderly, approximately 42.4–56.3% did not reverse their frailty by following the exercise program [27,28]. Fortunately, low-intensity exercises are well tolerated and have good adherence among the elderly, as well as a low risk of adverse events [29]. Therefore, a critical issue arises: How can low-intensity exercise also be effective in improving the frailty of frail elderly?

As a result, we hypothesized that eight-form Tai Chi requires far less physical ability and intensity [30]. Eight-form Tai Chi has been shown to improve lower limb muscle strength and gait speed in the frail elderly [31]. Therefore, we hypothesized that a hybrid exercise program incorporating eight-form Tai Chi and strength and endurance training could improve the health of the frail elderly. Additionally, because prior research has shown that appropriate exercise modalities can improve the exercise behavior of the elderly [32], exercise programs incorporating Tai Chi may be more effective at increasing exercise motivation in the frail elderly. Therefore, aiming to improve physical fitness and reverse frailty in the frail elderly, we attempted to integrate eight-form Tai Chi and a strength and endurance training program.

Explainable artificial intelligence (XAI) has been receiving increasing attention, especially in the healthcare field [33,34], as the interpretability involves making the system easier to understand and, therefore, to trust. The key to improving the efficacy is to use targeted interventions based on different levels of physical fitness. However, because of the complex mechanism of physical fitness, people always take a long time to choose a suitable exercise program, and subtle individual differences may result in completely different exercise programs. Therefore, we used artificial intelligence (AI) models to simulate the clinical treatment process. We used the initial physical fitness level and intervention type of the frail elderly as features to predict the frailty status of the elderly following the intervention, to assist in the design of exercise programs for the frail elderly.

In summary, this study hybridized eight-form Tai Chi and strength and endurance exercises to improve physical fitness and reverse the frailty of the elderly; furthermore, we tried to use 11 machine learning models to predict the frailty state based on initial physical fitness and different interventions. Based on previous studies, the following research hypothesis is proposed: (1) the hybrid exercise program combining eight-form Tai Chi, strength, and endurance exercises can improve physical fitness and reverse frailty; (2) the initial physical fitness and exercise program can predict whether frailty will be reversed in the frail elderly.

## 2. Materials and Methods

### 2.1. Participants

Since the analysis method of this study was entirely based on AI models, unlike statistical design of experiments, machine learning models require a larger sample size to train the model and prevent model overfitting. Therefore, 243 participants were enrolled by distributing study-related explanatory letters to community service centers for the elderly in Changchun, China. The following inclusion criteria were used to ensure the safety of the procedures and to avoid bias in the results: older people aged 65 years or older who gave their informed consent in the community were eligible.

Inclusion criteria: Participants meeting the following criteria were included in this study: (1) a Fried frailty phenotype score of 3–5 points; (2) the ability to walk for at least six minutes without assistance; (3) no other training in the preceding year.

Exclusion criteria: Participants meeting the following criteria were excluded: (1) those who had neurological diseases in the last 6 months; (2) persistent joint pain and severe muscle and bone injury to the extent that they were unable to walk normally; (3) history of mental illness or serious psychological problems resulting in an inability to follow any commands; and (4) participation in other training programs regularly during the study period.

The sample size was calculated based on a previous study of a multicomponent exercise program in the frail elderly, with effect size 0.53 and 80% power at an alpha level of 0.01 and a dropout rate of 20% [35].

### 2.2. Study Design

#### 2.2.1. Experimental Arrangement

**Figure 1:**
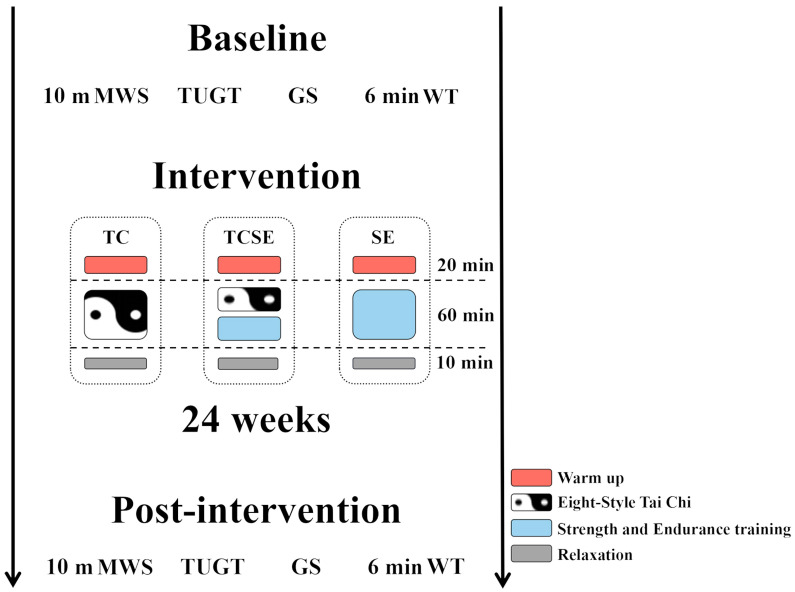
Experimental design. Participants were randomly assigned to the TC, TCSE, and SE intervention groups. Assessment of participants’ ten-meter maximum walking speed, TUGT, grip strength, and the six-minute walk test at baseline and after the 24-week intervention.

This study was a double-blind, randomized, controlled trial of exercise interventions for the frail elderly, as shown in Figure 1. The training instructors included one professional Tai Chi coach with a national Tai Chi instructor industry certification and one national social sports instructor with more than 8 years of practice instruction experience. The study participants were recruited from the community in Changchun, China, and the experiments were conducted on Monday, Wednesday, and Friday for 24 weeks. The subjects were randomly allocated to one of three groups: the eight-form Tai Chi intervention group (TC); eight-form Tai Chi, strength, and endurance training group (TCSE); and the strength and endurance training group (SE). All subjects signed an informed consent form in June 2018, following the approval of the study protocol by the Ethical Committee of Northeast Normal University (approval number: NC2018020401). Measurement and data collection were performed from 1 June to 30 December.

Subjects were asked to refrain from participating in any form of physical activity other than regular training.

#### 2.2.2. Intervention

Participants in the study completed three distinct intervention programs that included endurance training, strength training, and the traditional Chinese exercise eight-form Tai Chi. At the start of the intervention, all groups participated in 20 min of warm-up activities. TC had 60 min of Tai Chi training, while TCSE had 30 min of strength and endurance training and 30 min of Tai Chi training. SE received 60 min of strength and endurance training. All groups received 10 min of cool-down time before the end of the intervention. The details of the training are as follows:

(1) Tai Chi: The eight-form Tai Chi correspond to the eight forms of the twenty-four Yang-style Tai Chi. The training arrangement for eight-form Tai Chi consisted of two phases; the first and second phases lasted for 8 and 16 weeks, respectively. The TC group was scheduled to repeat the intervention two times during the first phase, whereas the TCSE group was scheduled to practice once. The second phase included three and two replicates for the TC and TCSE groups, respectively.
(2) Strength training: The strength training consisted of five exercises that activate the body’s major muscle groups [36]. Each training phase consisted of two lower-body exercises, including seated calf raises and hip adduction or abduction, as well as three upper-body exercises, including biceps curl, triceps pushdowns, and lateral pulldowns. The strength training in all exercises incorporated elastic exercise bands, with intensities determined by the color of the elastic exercise band. The training period was divided into three 8-week phases to optimize strength gain and muscle hypertrophy. The first phase focused on allowing participants to better adapt to high-intensity training of the second and third phases with light loads (40.0–60.0% of 1 RM) and high repetition counts (12–20), while also improving muscular strength and muscular endurance through the use of 2–4 sets of training. The second phase aimed to produce muscle hypertrophy and further increase the total muscle mass/fat ratio by gradually increasing the load to reach a maximum load of (60.0–80.0% of 1 RM), 5–12 repetitions, and 2–4 sets. To optimize strength development and further promote hypertrophy during the third phase, a higher load (70.0–85.0% of 1 RM) was used, 5–8 repetitions, and 2–4 sets. The SE group completed 4 sets of each movement, whereas the TCSE group completed half the number of sets, with a 2 to 3 min interval between sets.
(3) Endurance training: We used a heart rate monitor (Heart Rate Monitor, MYZONE MZ-3, China) to track the participants’ heart rate during training. Endurance training was performed on an indoor track using continuous walking. The target heart rate was individually adjusted from the initial measurement. The training intensity was gradually increased from 50.0% (first 12 weeks) to 80.0% (last 12 weeks) of the initial heart rate reserve [37]. The SE group completed 30 min of endurance walking and training, whereas the TCSE group completed 15 min. Each training session was accompanied by at least two medical personnel, and training was stopped immediately when the participant developed discomfort.

### 2.3. Assessment of Frailty

All subjective frailty levels were determined in this study using the Fried frailty criteria [4], with the elderly being classified as frail when three or more of the following five phenotype criteria were met:

(1) Unintentional weight loss: Subjects were asked if they had lost more than 4.5 kg or 5.0% of their body weight in the past year without intentional weight loss.
(2) Self-reported fatigue: Subjects were asked how often they felt unable to walk and engaged in anything very strenuously more than twice a week.
(3) Grip strength: Subjects’ grip strength was measured using a calibrated Jamar Hydraulic Hand Dynamometer (model SH5001, Saehan Corp, Masan, Korea, 2017); three tries’ measurements were performed for each individual, and the best score was selected, while the grip was checked to see whether it was less than 26 kg for males and less than 18 kg for females.
(4) Walking speed: Subjects’ walking time of 10 m was measured. Elderly people whose walking speed was less than or equal to 1 m/s were categorized as frail.
(5) Low level of physical activity: The physical activity of subjects was assessed using the Physical Activity Scale for the Elderly in the Chinese population (PASE-C) [38]. The cut-off value for males of less than 383 Kcals per week and females of less than 270 Kcals per week were classified as people with low physical activity.

### 2.4. Assessment of Physical Fitness

Six research assistants assessed the physical fitness of the participants before and after the intervention. We used ten-meter maximum walking speed, TUGT, grip strength, and the six-minute walk test to assess the physical performance of the subjects and conducted split half reliability. We assessed reliability by calculating the split half reliability of subjects’ test scores at baseline. The results showed that all four test programs had good reliability (split half reliability: r = 0.79, *p* < 0.001). The ten-meter maximum walking speed was determined since it was significantly associated with changes in frailty [39] and has good validity in the elderly [40]. The TUGT is a simple test without specific equipment and has demonstrated good validity for agility (r = 0.63) [41]. Low grip strength as a phenotype of frailty in later life increases the risk of disability, death, and disease [42,43], and Syddall et al. also determined that grip strength was significantly associated with the markers of frailty [44] and has good validity in reflecting overall muscle strength (r = 0.69) [45]. Low cardiorespiratory fitness, poor endurance, and limited neuromuscular ability are all significant manifestations of frail elderly, and the six-minute walk test has good validity in indirectly assessing endurance quality (maximal oxygen uptake) in the elderly (r = 0.77) [46]. Here are the details of the tests:

(1) Ten-meter maximum walking speed: In a quiet test environment, participants completed two fifteen-meter walking tests as fast as they could, and the time taken from 2.5 to 12.5 m was recorded to ensure steady-state measurements. The best score was used for analysis.
(2) TUGT: Participants sat in a standard 45 cm-high chair, and when they heard a prompt from the research assistant, they stood up and walked forward 3 m around the obstacle as fast as they could, then returned to sit down.
(3) Grip strength test: Grip strength was evaluated using a calibrated Jamar Hydraulic Hand Dynamometer (model SH5001, Saehan Corp, Masan, Korea, 2017). Participants completed three grip strength tests in a standing position, and the best result was taken as the test result.
(4) Six-minute walk test: The six-minute walk test was used to assess the endurance qualities of the participants. Subjects completed the test in an enclosed, flat, 30 m promenade. Markers were placed every three meters along the promenade, and turning points were placed at each end of the promenade. Subjects were instructed to complete the maximum distance possible in the promenade.

Requirements for each motor ability test: first, the experimental subjects should wear sportswear and comfortable sports shoes and complete the experiment independently; second, the subjects should familiarize themselves with the experiment route in advance; third, the participants should perform a warm-up to avoid unnecessary injuries; finally, if the participants feel physical discomfort, discomfort related to body position, or environmental discomfort, they should report this. The experiment can be terminated at any time.

### 2.5. Data Analyses

A total of 10 commonly used machine learning classification models were utilized to predict the post-experimental frailty status of the frail elderly. The ten-meter maximum walking speed, grip strength, TUGT, six-minute walk test, and three intervention types before the intervention were used as features, and whether the subjects were frail or not after the intervention were used as labels to construct the dataset. The performance of evaluation metrics was determined based on the accuracy, recall, prediction, and area under the curve (AUC). To accurately evaluate the model performance, we repeated the stratified 10-fold cross-validation 100 times. Classical machine learning modeling was first performed, using the Extra Tree Classifier (ETC), Gradient Boosting Classifier (GBC), k Neighbors Classifier (KNN), Linear Discriminant Analysis (LDA), Logistic Regression (LR), LightGBM Classifier (LGBM), Decision Tree (DT), Support Vector Classifier (SVC), Random Forest Classifier (RF), and XGBoost Classifier (XGB). The three models with the best performance in this dataset were then selected for stacking modeling. In this study, stacking was performed by combining multiple classifiers generated by different learning algorithms $L_{1},\ldots ,L_{n}$ on a single dataset *S*, which consisted of examples $S_{i}$ = ($x_{i}$, $y_{i}$), where $x_{i}$ denotes feature vectors and $y_{i}$ corresponds to the classifications. First, a set of base-level classifiers $C_{1}$, $C_{2}$, and $C_{3}$ was generated, where $C_{i}$ = $L_{n}$ (*S*). In the second phase, a meta-level classifier was learned, which contained the outputs of the base-level classifiers. To generate a training set for learning the meta-level classifier, a cross-validation procedure in which each of the base-level learning algorithms was applied to almost the entire dataset was carried out, leaving one example for testing, as: $\forall i=1,\ldots ,n:\forall k=1,\ldots ,N:C_{k}^{i}=L_{k}\left(S-s_{i}\right)$. We then used the learned classifiers to generate predictions for $S_{i}$, as Equation (1):

(1) $${\hat{y}}_{i}^{k}=C_{k}^{i}\left(x_{i}\right);$$

here, the meta-level dataset consists of examples of the form ((${\hat{y}}_{i}^{1}$,⋯, ${\hat{y}}_{i}^{n}$), $y_{i}$), where the features are the predictions of the base-level classifiers and the class is the correct class of the example at hand.

To identify which feature contributed most to predict the change of the frail state, we calculated Shapley additive explanation (SHAP) values [47]. SHAP is a game theoretic approach to explain the output of any machine learning model; SHAP values can quantify the contribution that each feature brings to the prediction made by the model, as Equation (2):

(2) $$\phi_{j}=\sum_{S_{F}\subseteq F\backslash \left\{j\right\}}\frac{|S_{F}\left|!(|F|-\right|S_{F}\left|-1\right)!}{|F|!}\left[f_{S_{F}\cup \left\{j\right\}}\left(x_{S_{F}\cup \left\{j\right\}}\right)-f_{S_{F}}\left(x_{S_{F}}\right)\right],$$

where *x* is the values of the input features, *j* is a certain feature (out of total features *F*), $S_{F}$ indicates all possible subsets without feature *j*, and $|S_{F}|$ is the dimension of $S_{F}$. To compute this effect, a model $f_{S_{F}\cup \left\{j\right\}}$ was trained with feature *j* present, and another model $f_{S_{F}}$ was trained with feature *j* withheld. In this study, the SHAP “TreeExplainer” algorithm was used to determine the most important feature in predicting the change of frail state.

### 2.6. Statistical Analyses

Statistical analyses were performed on TUGT, ten-meter maximum walking speed, grip strength, and the six-minute walk test. Data distributions were analyzed using the Shapiro–Wilk test, and non-normally distributed data were transformed using the Log transform and expressed as Mean ± SD. The comparison of demographic variables used one-way ANOVA for continuous data and the chi-squared test for categorical data. Two-way repeated measures analysis of variance (ANOVA) was performed to determine significant main effects and interaction effects. The Bonferroni post hoc test was used for multiple comparisons. The level of statistical significance was set at *p* < 0.05. All data analyses and statistical analyses in this study were performed in Python 3.7.1.

## 3. Results

After a rigorous screening process carried out of 234 enrollees, 174 participants (102 females and 72 males) met the selection criteria. The final 150 participants completed the study (84 females and 66 males). The reasons for not completing the training included voluntary dropout (*n* = 9) and termination of training due to illness (*n* = 15). The baseline information and various physical fitness values are shown in Table 1. These subgroups did not differ from each other in relevant demographic or performance measures.

**Figure 2:**
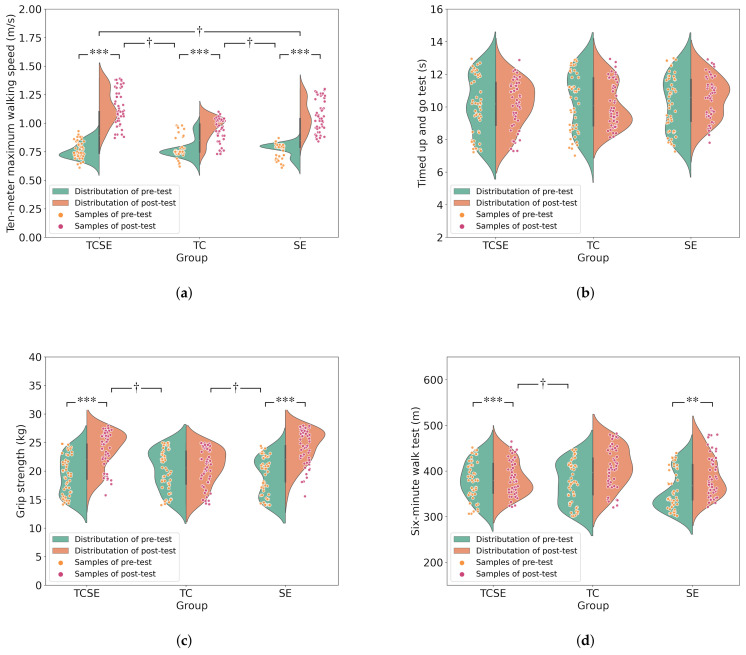
The figure is the scatter plot with the addition of a rotated kernel density plot on each side; it shows the physical ability of the frail elderly at baseline and post-intervention. (**a**) The ten-meter maximum walking speed, (**b**) TUGT, (**c**) grip strength, and (**d**) six-minute walk test. TC indicates Tai Chi group; TCSE indicates Tai Chi, strength, and endurance group; SE indicates strength and endurance group. ** *p* < 0.01, *** the significance of intragroup differences (*p* < 0.001), † the significance difference among groups (*p* < 0.05).

At the end of the experiment, the researchers reassessed the subjects’ frailty status. The results showed that 69 (46.0%) improved from a frail state to a non-frail state, including 14 (20.3%) in the TC group, 31 (44.9%) in the TCSE group, and 24 (34.8%) in the SE group. In addition, two-way repeated measures analysis of variance (ANOVA) on ten-meter maximum walking speed, TUGT, grip strength, and the six-minute walk test is shown in Figure 2 and Table 2.

The two-way repeated measures ANOVA results showed that the group effects of ten-meter maximum walking speed, grip strength, TUGT, and the six-minute walk test before the intervention were not significant. There was a significant interaction effect of group × time in ten-meter maximum walking speed, grip strength, and the six-minute walk test. Post hoc tests showed that after 24 weeks of intervention, subjects in the TCSE group showed the greatest significant improvements in ten-meter maximum walking speed (*p* < 0.05) and the six-minute walk test (*p* < 0.05) compared to the TC group and SE group. The improvement in grip strength in the TCSE group (4.29 kg) was slightly less than that in the SE group (5.16 kg). There was neither a significant main effect nor a significant interaction effect for TUGT in subjects.

We used two states, frailty and non-frailty, as labels, and the pre-intervention TUGT, 10 m maximum walking speed, grip strength, the six-minute walk test, and three intervention types as features to train the models for predicting frailty states after intervention. For the 10 models, the performance is shown in Table 3. The three classification models, Random Forest, Gradient Boosting, and K Nearest Neighbors, were then stacked to construct the first-layer model, and the dataset was input into the model to construct the superfeatures; the superfeatures and labels were input as data to the second layer, which utilized LR as the model. The results showed that our stacking model obtained the best accuracy of 67.8 ± 11.5% and the best F1-score of 71.3 ± 10.8%. However, the Gradient Boosting Classifier had the best precision results compared with the other algorithms (70.0 ± 12.2%). Furthermore, we assessed the accuracy (55.6 ± 13.3%) of the surrogate dataset, and the performance of stacking was significantly better than the chance level.

**Figure 3:**
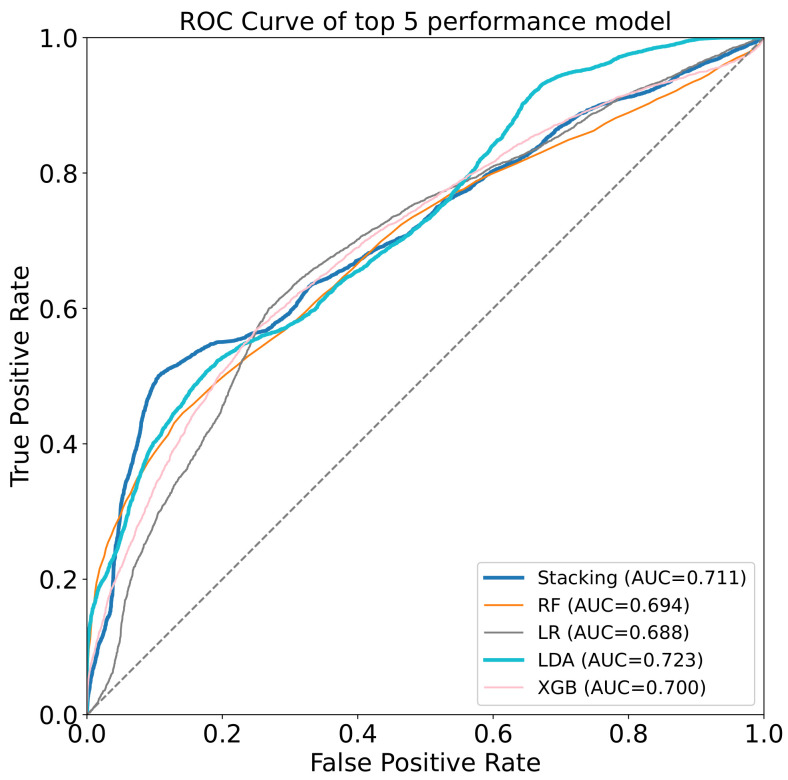
The ROC curve of the top-5 performance models. RF denotes Random Forest Classifier; LR denotes Logistic Regression; LDA denotes Linear Discriminant Analysis; XGB denotes XGB Classifier.

**Figure 4:**
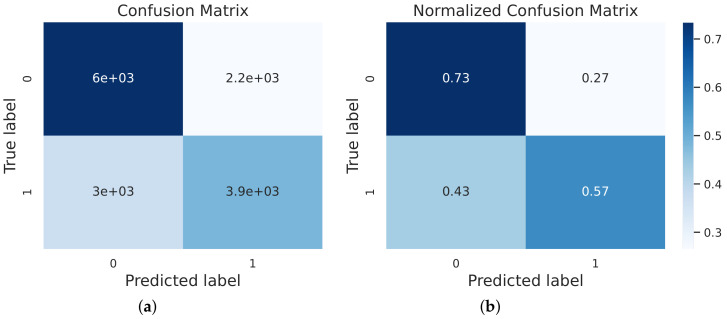
The confusion matrix of stacking. (**a**) confusion matrix; (**b**) normalized confusion matrix. The plots reveal the performance of identifying the various states of frailty; among them, “frail” had a better result.

Furthermore, Figure 3 shows the receiver operating characteristic (ROC) curve of the model for evaluating the model performance; the horizontal coordinate indicates the false positive rate; the vertical coordinate indicates the true positive rate; the area under the curve (AUC) value of the stacking model is 0.711. The output of the confusion matrix indicates the model’s prediction of the weak state, and this study simultaneously visualized the confusion matrix and the normalized confusion matrix to better visually represent the predicted performance. In each row of Figure 4a, the confusion matrix represents the predicted categories, and each column represents the actual labels, while the elements of Figure 4b are the normalized confusion matrix subdiagonal representing the average accuracy of the model’s predictions for each category, which were 57.0% and 73.0%, respectively.

**Figure 5:**
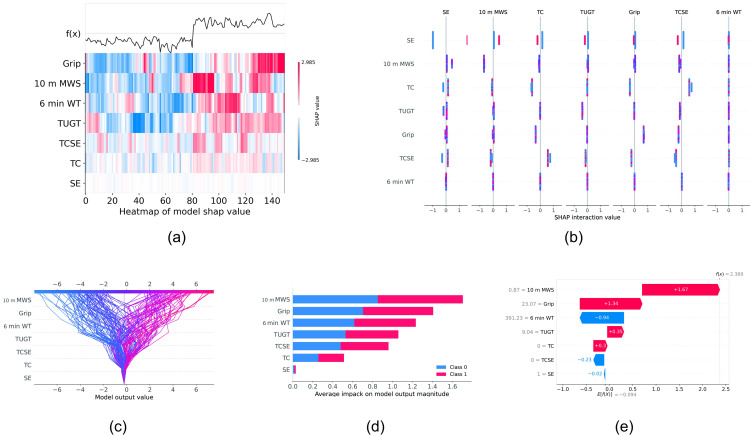
A summary plot of the SHAP values for each feature. The y-axis denotes the different features. (**a**) Indicates the heat map of different feature SHAP values, where the *x*-axis indicates the sample sequence, red indicates positive influence, and blue indicates negative influence; the darker the color, the greater the degree of influence. *f*(*x*)*p* indicates the output (before activation function). The figure shows that the most important feature has a positive effect if the output is positive and also indicates that there is a clear boundary (threshold) in the features. (**b**) denotes the interaction SHAP values of different features, revealing the interaction effects between different features. (**c**) denotes an overview of the features that are most important for a model and how the model obtained the output of every feature for every sample, where the *x*-axis denotes the model-predicted values. (**d**) denotes the average absolute SHAP value of different features; the *x*-axis denotes the average SHAP value. The ten-meter maximum walking speed contributed the most to predicting the frailty state. (**e**) represents features contributing to pushing the model output from the base value of a single sample, where the *x*-axis represents the SHAP value. In this case, the ten-meter maximum walking speed contributed the most positive effect, and the six-minute walk test contributed the most negative effect for predicting the frailty state.

The contribution of each feature calculated from the SHAP value is shown in Figure 5. The SHAP value shows the degree of contribution of each feature to the model performance. Among the four physical fitness, ten-meter maximum walking speed had the greatest contribution to the prediction of each sample, and among the three intervention types, the TCSE intervention had the greatest contribution. As Figure 5b shows, most features could interact with each other. Obviously, the best feature combination can be found by examining the performance of all combinations of features, which indicates better performance.

## 4. Discussion

To the best of our knowledge, this is the first study to propose a hybrid exercise strategy that incorporates traditional Chinese health practices and a strength and endurance training program for the frail elderly and uses artificial intelligence to predict frailty as a clinical assistant. The findings showed that after 24 weeks of training, all groups showed varying degrees of physical improvement in strength, speed, and endurance. The hybrid exercise program was the group demonstrating the most improvement in the 10 m maximum walking speed and the six-minute walk test. Additionally, the hybrid exercise program demonstrated the best results in reversing frailty, with 62.0% of the frail elderly recovering from a non-frail state. Meanwhile, we innovatively introduced artificial intelligence to obtain a 67.8% average accuracy by simulating clinical application scenarios using pre-intervention exercise capacity and the intervention protocol as input model features and frailty reversal as the output.

Frailty is not a contraindication to exercise, but rather, a reason to recommend it. As previously stated, frailty is the result of a combination of physiological systems: On the one hand, a decline in cardiopulmonary function and skeletal muscle content is the primary cause of weakness, with a 30.0–40.0% reduction in skeletal muscle content between the ages of 50 and 80 [48], and numerous studies have demonstrated that exercise is an effective way to improve skeletal muscle mass and cardiorespiratory fitness [49,50]. Thus, a combination of interventions can affect frailty states [27,51,52]. Additionally, we found that ten-meter maximum walking speed and grip strength contributed the most to the feature and were highly identifiable: the frail elderly with a greater ten-meter maximum walking speed and grip strength may more easily reverse frailty. As frailty is defined clinically as shrinking, exhaustion, inactivity, slowness, and weakness [4], an increase in ten-meter maximum walking speed and grip strength may make the most contribution. Additionally, because the feature of SE did not contribute to the prediction, the model utilized a one-hot encoder, if TCSE = 0, TC = 0, and SE = 1, and the input can be regarded as (0, 0), which equals (0, 0, 1).

After 24 weeks of receiving different combinations of exercise program interventions, subjects in the TCSE group demonstrated the greatest improvement in ten-meter maximum walking speed, as 0.38 m/s (TCSE) > 0.29 m/s (SE) > 0.19 m/s (TC), and the six-minute walk test, as 36.18 m (TCSE) > 32.86 m (SE) > 5.61 m (TC), and the time × group interaction had significant effect. For the reasons that the hybrid exercise program had the best results, first and foremost, a multi-component exercise program is an effective approach, since it enables frail patients to take advantage of different exercise modalities to compensate for their deficiencies [16]. Second, the characteristics of Tai Chi, as a low-to-medium intensity exercise [53], can help practitioners improve their neuromuscular control [54], and it has additional benefits for lower-limb muscle strength, such as increasing body stability when walking, etc. [16]. Therefore, after integrating endurance training with walking as an exercise tool, the relatively slow movement characteristics of Tai Chi are compensated by continuous dynamic movement, which is more conducive to improving walking ability in a real environment. Finally, because Tai Chi enhances the function of the respiratory system, the combination of strength and endurance training might significantly improve cardiorespiratory endurance in frail elderly people [17]. Thus, subjects in the TCSE group had the most significant improvement in ten-meter maximum walking speed and the 6 min walk test.

In this study, the improvement in grip strength levels in the TCSE group was slightly less than in the SE group (5.16 kg (SE) > 4.29 kg (TCSE)). Participants in the TC group exhibited a small increase in grip strength, but there was no significant difference. However, as previously stated, Tai Chi mainly focuses on improving lower-limb muscular mobility [54], and the TCSE group, while including the training content of the SE group, trained the upper limb less intensely than the SE group at the same training load, resulting in a slightly smaller improvement in grip strength level in the TCSE group than in the SE group. Although the subjects in the TCSE group and SE group had improved grip strength, we believe that this may only be a local side effect and did not demonstrate an overall strength improvement in the subjects.

Surprisingly, the frail elderly did not demonstrate significant differences in TUGT across groups following the intervention, although there was a trend toward improved overall TUGT. This finding is consistent with the findings of numerous research [55,56]. Although some studies have shown improved TUGT in the frail elderly [35,57], we believe that there are several reasons for the lack of improvement in our results. First, because the subjects in this study were frail elderly people, low intensity was used in designing the exercise program, and the training contents focused more on endurance and strength, and less on improving agility. Second, endurance and strength exercises consist of mainly self-weight and small load exercises, which may have a negligible effect on agility. Third, although Tai Chi improves agility, it is possible that even with a mixed exercise program, the improvement in agility quality remains restricted due to the limited physiological ability of the frail elderly. Therefore, the level of TUGT was not improved in the frail elderly groups.

Although our study yielded promising results, there are several problems. First, our study only controlled for training time, not intensity. Second, we found that while the hybrid exercise program approach improved strength and endurance, there are still significant limitations in terms of agility quality, and further research may be needed in the future to address this issue. Third, our intervention program lacked exercise for the muscles involved in grip strength. Fourth, the improved grip strength in subjects may only be the result of a localized side effect, and TUGT may not be a very desirable evaluation method. Finally, we believe that the frail elderly may have a more profound potential relationship with frailty reversal. Based on the relationship between frailty reversal, the initial state of the frail elderly, as well as the intervention program, we plan to integrate the data of the same experimental design to build a more complete feature system and achieve more accurate medical assistance.

## 5. Conclusions

Our findings indicate that a hybrid exercise program of eight-form Tai Chi, strength, and endurance can more effectively improve physical fitness and reverse frailty in the frail elderly, and the improvement of motor ability in the elderly is highly dependent on the training content. Additionally, our stacking model enables the prediction of frailty in the elderly. Our constructed model performed significantly better in terms of prediction.

## Figures and Tables

**Table 1:** Baseline demographic characteristics of the participants.

Items	TC ^1^ (*n* = 50)	TCSE ^2^ (*n* = 50)	SE ^3^ (*n* = 50)	*p*-Value
Sex (male/Female)	25/25	20/30	21/29	0.566
Age (years)	75.60 ± 1.92	76.31 ± 2.07	76.59 ± 2.58	0.076
Body mass (cm)	165.64 ± 8.30	164.51 ± 8.04	162.26 ± 12.60	0.228
Stature (kg)	62.95 ± 6.33	62.10 ± 6.32	64.23 ± 6.40	0.248

^1^ Tai Chi group; ^2^ Tai Chi, strength, and endurance group; ^3^ strength and endurance group.

**Table 2:** Two-way repeated measures ANOVA results for each group at baseline and 24 weeks for the test metrics.

Parameters	TC ^1^ (*n* = 50)		TCSE ^2^ (*n* = 50)		SE ^3^ (*n* = 50)		Group × Time ^#^
	Baseline	24 Weeks	Baseline	24 Weeks	Baseline	24 Weeks	*p*-Value
10 m MWS (m/s)	0.77 ± 0.08	0.96 ± 0.10 ^†,^***	0.76 ± 0.07	1.14 ± 0.15 ^†,^***	0.77 ± 0.06	1.06 ± 0.14 ^†,^***	<0.05
TUGT (s)	10.32 ± 1.81	10.18 ± 1.38	10.09 ± 1.80	10.11 ± 1.47	10.19 ± 1.72	10.52 ± 1.30	0.542
Grip Strength (kg)	20.13 ± 3.52	20.45 ± 3.35 ^†^	19.33 ± 3.47	23.62 ± 3.25 ^†,^***	18.93 ± 3.44	24.09 ± 3.08 ^†,^***	<0.05
6 min WT (m)	380.05 ± 41.86	385.66 ± 38.59 ^†^	372.72 ± 46.55	408.90 ± 46.45 ^†,^***	358.60 ± 43.88	391.46 ± 44.76 **	<0.01

All data are expressed as Means ± SD; ^#^ analysis of two-way repeated measures ANOVA; ^†^ significant difference between groups (*p* < 0.05); ** *p* < 0.01, *** significant difference between baseline and post-intervention (*p* < 0.001). ^1^ Tai Chi group; ^2^ Tai Chi, strength, and endurance group; ^3^ strength and endurance group.

**Table 3:** Baseline demographic characteristics of the participants.

Models	Accuracy	Precision	Recall	F1
SVC ^1^ (%)	60.0 ± 12.4	61.9 ± 11.1	70.9 ± 19.4	65.0 ± 13.2
Logistic Regression (%)	62.3 ± 12.3	65.7 ± 12.6	69.8 ± 16.8	66.7 ± 11.9
LDA Classifier ^2^ (%)	63.8 ± 11.8	66.6 ± 11.7	67.1 ± 16.3	65.9 ± 12.2
LGBM Classifier ^3^ (%)	64.2 ± 11.3	67.3 ± 12.3	68.5 ± 16.2	67.3 ± 11.6
Decision Tree (%)	64.4 ± 11.7	68.2 ± 12.9	66.7 ± 16.8	66.4 ± 12.6
Extra Tree Classifier (%)	65.7 ± 11.5	67.4 ± 11.2	71.6 ± 15.9	68.5 ± 11.3
XGB Classifier ^4^ (%)	65.7 ± 11.3	69.0 ± 13.0	69.0 ± 15.8	67.3 ± 12.3
KNN Classifier ^5^ (%)	66.4 ± 11.4	67.7 ± 11.4	**75.7 ± 15.4**	71.0 ± 10.5
GDB Classifier ^6^ (%)	67.2 ± 11.7	**70.0 ± 12.2**	72.0 ± 15.8	70.2 ± 11.6
RF Classifier ^7^ (%)	67.4 ± 10.9	69.7 ± 11.5	71.6 ± 16.2	69.6 ± 11.2
**Stacking (%)**	**67.8 ± 11.5**	69.4 ± 11.8	74.5 ± 15.9	**71.3 ± 10.8**

^1^ Support Vector Classification, ^2^ Linear Discriminant Analysis, ^3^ LightGBM Classifier, ^4^ XGBoosting Classifier, ^5^ K Neighbors Classifier, ^6^ Gradient Boosting Classifier, ^7^ Random Forest Classifier. We compared the performance of different models, and the stacking model showed the best performance in accuracy, precision, recall, and F1-score. The stacking model utilized Random Forest, Gradient Boosting, and K Nearest Neighbors as the first layer and Logistic Regression as the second layer.

## Data Availability

The datasets used and/or analyzed during the current study are available from the corresponding author upon reasonable request.
